# Natural *Brucella melitensis* Infection and Rev. 1 Vaccination Induce Specific *Brucella* O-Polysaccharide Antibodies Involved in Complement Mediated *Brucella* Cell Killing

**DOI:** 10.3390/vaccines10020317

**Published:** 2022-02-17

**Authors:** Shubham Mathur, Menachem Banai, Dani Cohen

**Affiliations:** 1 Department of Epidemiology and Preventive Medicine, School of Public Health, Sackler Faculty of Medicine, Tel Aviv University, Tel Aviv 6997801, Israel; shubhamm@mail.tau.ac.il; 2 Kimron Veterinary Institute, Beit Dagan 5025001, Israel; menachemba48@gmail.com

**Keywords:** *Brucella melitensis*, serum, bactericidal activity, O-polysaccharide, *Brucella* OPS IgG antibodies

## Abstract

Vaccination against brucellosis using live attenuated strains is the primary approach in protecting livestock against the disease through a strong cellular immune response. Attenuated vaccine strains also induce serum anti-*Brucella* antibodies, mostly against *Brucella* O-polysaccharide, but their role in protection against the disease remains unclear. In this study, we show that *Brucella* OPS serum antibodies after vaccination or natural infection could kill *Brucella* in vitro as shown by the serum bactericidal activity (SBA) assay. We used serum samples of Rev. 1 vaccinated sheep that were negative or positive for *Brucella* OPS antibodies by either one of complement fixation test (CFT), microplate agglutination test (MAT) and ELISA, or sera of naturally infected sheep positive by CFT. We found a significant increase in the killing ability of sera 30 days after intraocular vaccination with Rev. 1 as compared with pre-vaccination. SBA was significantly higher in sera containing *Brucella* OPS IgG antibodies in comparison with sera lacking such antibodies (*p* < 0.001 against 16M & Rev. 1 strains). All 10 sera of convalescent sheep demonstrated significant killing ability against the 16M *B. melitensis* field strain. Specific OPS antibodies participate in the in vitro complement mediated *Brucella* killing suggesting a potential role in protection against the disease through this mechanism and relevance of developing OPS-based *Brucella* vaccines.

## 1. Introduction

Brucellosis severely affects animal and human health [1]. *Brucella* species belong in the Gram-negative group, partly classified according to their natural affiliation to distinct animal species. To date, 12 species have been validly published but only three, *B. melitensis*, *B. abortus* and *B. suis* associated with small ruminant, bovid and swine brucellosis, respectively, are the most distinct human pathogens. Moreover, small ruminants that are natural hosts of *B. melitensis,* establish a critical vehicle in transmitting the disease to humans, and partly to bovines. *Brucella* species cause abortion storms in their natural animal hosts followed by excessive shedding of the bacteria via contaminated placenta, fetus membranes and fluids, which leads to spreading of free *Brucella* organisms in the farm and environment [2]. Brucellosis is transmitted to humans through their contact with infected animals or with contaminated material such as aborted placenta or following consumption of contaminated raw milk and dairy products. In humans, brucellosis is frequently confused with flu-like symptoms but may exacerbate into complications such as endocarditis, meningitis and osteo-articular or epididymo-orchitis manifestations, and may rarely be lethal [3].

Controlling the disease in the livestock population is the most effective approach in preventing human cases and reducing the overall burden of disease in both the animal and human health systems. An ideal brucellosis control program consists of prophylactic vaccination of the livestock with live attenuated strains, smooth *B. abortus* S19 strain or rough *B. abortus* RB51 in cattle and *B. melitensis* Rev. 1 in small ruminants, respectively [4,5,6]. A test and cull policy after serological confirmation might complement successful eradication campaigns where possible, as owner compensation is mandatory in such cases [7]. Such a dual control policy has been implemented successfully in Israel throughout the years, resulting in minimizing the problem of *B. melitensis* [8,9] but failure to sustain the program due to budgetary concerns has hampered these achievements.

The host first encounters *Brucella* by mediating an innate bactericidal response against organisms residing extracellularly with a successive development of an effective protective cellular response [10]. The live vaccine strains resembling their field strains in engaging the host immune system only after entrance establish a Th1 immune response involving humoral and cell-mediated B and T cells [11,12,13]. Importantly, these vaccines protect against abortion and therefore reduce bacterial spread in the farm, thereby decreasing the economic and health burden in the farm. However, despite achieving effective vaccination coverage with the live strains, Israeli dairy herds and many small ruminants have been consistently infected with *B. melitensis* field strains inferring on lack of protection against bacterial entrance to the host [9].

Here, we hypothesized that anti-*Brucella* OPS antibodies might fulfil the missing fragment in sustaining herd protection. Previous mice experiments have resulted with controversies regarding the specific contribution serum antibodies might play in host protection against *Brucella* challenges [11,14,15,16,17,18]. In light of the unresolved disagreement, we have revisited the question whether anti-*Brucella* OPS serum antibodies could be protective against brucellosis through complement mediated killing of *Brucella* cells during the extracellular stage of infection.

In this study we show that anti-*Brucella* OPS antibodies developed post-vaccination or following natural field infection kill *Brucella* in vitro as revealed by the serum bactericidal activity assay [19,20]. These findings open new horizons to developing a successful *Brucella* OPS-conjugate vaccine as recently suggested by Bundle and McGiven [18,21,22] that could induce high levels of such functional antibodies.

## 2. Material and Methods

### 2.1. Bacteria

*B. melitensis* 16M, a smooth virulent *Brucella* type strain (Received in the laboratory from INRA, (Institut National de la Recherche Agronomique (INRA) 75007 Paris, France), and *B. melitensis* vaccine strain Rev. 1 Elberg, passage 101, 1970, recommended by the WHO as an authentic vaccine strain for commercial production for sheep and goats (received directly from Dr. Elberg as a freeze-fried powder) were used in the study. The two strains are stored as glycerol frozen suspensions at the Israeli OIE and FAO Reference Laboratory for Brucellosis, Kimron Veterinary Institute, Israel. For an experimental use, these frozen suspensions were reconstituted on Tryptic Soy agar plates supplemented with Serum-Dextrose solution [23].

### 2.2. Serum

Sheep vaccination and monitoring against brucellosis is mandatory in Israel (Small ruminant health–Veterinary Services (in Hebrew), Last up-dating 15.03.2021 Available online: https://www.gov.il/he/departments/general/sheep-health-veterinary-services (accessed on 9 February 2022). The Israeli Veterinary Services employ a single, lifelong dose of an ocular Rev. 1 vaccine, at 1–2 × 109 CFU/per animal. Vaccination is restricted to female ewe-lambs at ages 3–6 months. Males are not vaccinated but used as sentinel in monitoring herds for the disease. Animals which are intended for trade are checked serologically by Complement Fixation Test (CFT) and Microplate Agglutination Test (MAT). Otherwise, in case of suspected disease, all adult females are tested serologically by CFT.

Sheep serum samples from 2 different flocks at different time periods were taken by staff personnel of the Israeli Veterinary Services during routine surveillance and control activities and collected into Vacuette tube for serum separation.

Serum samples from infected sheep were collected from animals in a bacteriologically confirmed infected flock. Ocular Rev. 1 vaccination of female ewe-lambs was conducted as part of the Israeli Veterinary Services vaccination program in one flock known to be brucellosis free, and sera samples were obtained from the animals before vaccination at day 0, and after vaccination at day 15 and day 30. All serum samples were stored at 4 °C until their use. Anti-*Brucella* OPS serological titers were tested by complement fixation test (CFT, titer conversion to IU = ×1.6) and microplate agglutination test (MAT, titer conversion to IU = ×1.905) [24].

### 2.3. Indirect ELISA for Brucella–OPS Antibodies

Besides conducting the standard serological tests, sera obtained in the vaccine experiment were evaluated for presence of anti-*Brucella* OPS antibodies by ELISA using the commercially available kit ID Screen^®^ Brucellosis Serum Indirect Multi-Species (IDVET, Amman, Jordan). Results were expressed in OD values measured at 450 nm as indicated by manufacturer.

### 2.4. Serum Bactericidal Activity

Fresh bacterial cultures of *B. melitensis* 16M and *B. melitensis* Rev. 1 passage 101 Elberg strain were grown in Tryptic Soy broth (TSB), for 2 days at 37 °C in presence of 5% CO_2_ atmosphere. Bacterial suspensions were centrifuged at 10,000× *g* for 10 min and cell pellets were suspended in PBS-containing 0.5 mM MgCl_2_ and 0.15 mM CaCl_2_, and cells were serially diluted to reach a cell concentration of 10^4^ CFU per ml. Each serum sample was tested in four separate experiments, in duplicates using Oswald et al. (1990) method with minor modifications [25]. Shortly, sheep serum was heat-inactivated at 56 °C for 30 min, and 50 µL were mixed with 30 µL of the master *Brucella* dilution in 96-well flat-bottomed polystyrene microtiter plates (Nunc™ Thermo scientific, Rochester, NY, USA). Cells were incubated in presence of 5% CO_2_ atmosphere, keeping the plate rotated at 100 rpm for 30 min at 37 °C, which allowed *Brucella* cells to interact with antibodies. After 30 min, 40 µL of human complement (Quidal, San Diego, CA, USA) was added to the *Brucella*-serum reaction mixture and incubation at 37 °C was continued for additional 1.5 h. Then, a 25 µL aliquot from each well (estimated number of 62 CFU in the untreated suspension) was plated in duplicates on Serum-Dextrose tryptic soy agar plates and incubated for 2 days at 37 °C with 5% CO_2_ for CFU counting. Two control experiments were conducted, a *Brucella* cell suspension incubated in presence of buffer without serum and a *Brucella* cell suspension incubated in presence of complement but without serum, respectively. Because results showed only a minimal cell killing by the two systems, around 2–3% (data not shown), we used the complement reaction mixture as our negative control in further calculations [11,26]. The percentage of cell killing (killing efficiency) was calculated as follows.

$$\%K=100x\left\{1-\left(\frac{mean\;no.of\;CFU\;after\;incubation\;with\;serum\;and\;complement}{mean\;no.\;of\;CFU\;after\;incubation\;only\;with\;complement\;}\right)\right\}$$

### 2.5. Statistical Analysis

Graphpad Prism (Graphpad Prism. Available online: https://www.graphstats.net/graphpad-prism (accessed on 19 July 2021) was used for statistical analysis. Bonferroni’s multiple comparison tests was used to analyze significance of differences of *Brucella* cell killing between serum samples of non-vaccinated and vaccinated ewes according to post-vaccination time periods two ways ANOVA unweighted analysis was employed (Bonferroni post hoc test was used to analyze significance of differences between serum activity without or in presence of anti-*Brucella* OPS antibodies. Unpaired *t*-test was used to identify the Brucella killing by post-infection serum samples.

## 3. Results

### 3.1. Serology and Serum Bactericidal Activity

#### 3.1.1. Serology and Serum Bactericidal Activity (SBA) of Post-Vaccination Sera of Flock 1

*B. melitensis* Rev. 1 strain vaccination is used routinely in Israel in young replacement ewe-lambs as a full dose (1–2 × 10^9^ cfu) ocular application. This vaccination approach was introduced in the 1980s as a method of differentiating infected from vaccinated animals (DIVA) solution to minimize post-vaccine humoral antibody titers [7]. About 40% of the animals have been found negative in the MAT and CFT tests (unpublished data). We chose this platform to specifically test our hypothesis regarding development of complement mediated anti-*Brucella* OPS serum bactericidal activity (SBA) of antibodies induced by vaccination. We used *B. melitensis* strain 16M, a type strain of the genus, and strain *B. melitensis* Rev. 1, Elberg passage 101, 1970, a reference vaccine type strain, as readout cells, addressing the potential attenuation of strain Rev. 1 in this pathway.

Three time periods were chosen, day 0 before vaccination, and 15 and 30 days post-vaccination, respectively. Day 0 expressing SBA before animal vaccination, e.g., in naïve animals with background serum components in comparison to post-vaccination time periods that anticipate contribution of serum-developed anti-*Brucella* OPS antibodies to *Brucella* cell killing.

As shown in Table 1, our initial experiment included four animals which were tested before and after vaccination with Rev. 1 as mentioned above. None had detectable antibody titers by both MAT and CFT before vaccination. Serum number 9914 was negative for anti-*Brucella* OPS by CFT and MAT during the whole experiment. In contrast, serum number 9912 had positive MAT titers on day 30 but was CFT-negative along this period, and serum numbers 9913 and 9921 that showed MAT titers at early days after vaccination (day 15) became responsive also in the CFT test at day 30. Cell killing of both strains increased according to the post-vaccination time period but the extent of cell killing between individual serum samples and time periods varied. SBA of the consecutive sera of 3 of 4 animals was significantly higher on days 15 and 30 post vaccination against both Rev. 1 and 16M strains (*p* < 0.05 16M; *p* < 0.01 Rev. 1, Bonferroni’s multiple comparison test). Serum number 9914 that lacked anti-*Brucella* OPS antibodies along the whole experiment showed nevertheless a strong SBA activity even at time 0 that is before vaccination. As shown in Supplementary Material Figures S1 and S2, this result was unexpected, as other naïve serum samples only killed *Brucella* cells at background levels assumably associated with nonspecific cross-reactive antibodies. Accordingly, one might link the higher SBA activity of serum sample 9914 with presence of non-*Brucella* reactive serum components.

**Figure 1:**
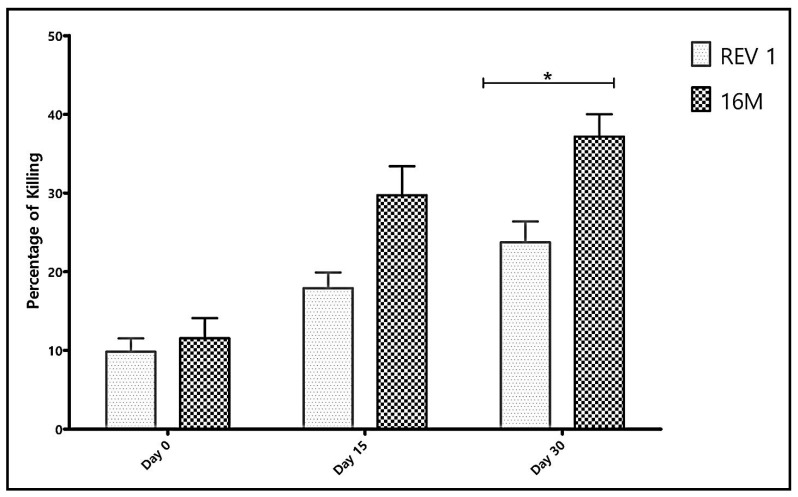
Arithmetic mean value of the combined percentages of *Brucella* cell killing of the four serum samples in correlation with post-vaccination time period. Percentage of cell killing is increased with post-vaccination time course (two-way ANOVA *p* < 0.001). Bonferroni post hoc test analysis of the mean values showed stronger killing of 16M in comparison to Rev.1 among all serum samples tested, being non-significant at day 0 (*p* > 0.05); and day 15 (*p* > 0.05) and turning out significant at day 30 (* *p* < 0.001), respectively. Bars stand for standard deviation.

Figure 1 summarizes the mean value of the collective SBA activities of the 4 sera samples against Rev. 1 and 16M strains at the 3 timings related to vaccination. Pre-vaccination naïve sera (day 0) had an SBA of around 10% cell killing against both *Brucella* strains, 16M and Rev.1. As post-vaccination time period increased, there was increased killing activity by the serum samples against both strains (*p* ≤ 0.0001, two-way ANOVA). The mean SBA was highest on day 30 post-vaccination with percentages of cell killing significantly higher against 16M than against Rev. 1 (*p* < 0.001, Bonferroni post hoc test). As depicted in Table 1, at this time after vaccination, 3 out of 4 and 2 out of 4 sera, had elevated IgM and IgG antibody titers against *Brucella* OPS, respectively.

#### 3.1.2. Serology and Serum Bactericidal Activity (SBA) of Post-Vaccination Sera of Flock 2

In an attempt to better understand the importance of antibody development to the extent of SBA, we performed a second experiment, using the same treatments as in the above but measuring antibody isotype titers by adding indirect ELISA (i-ELISA) to the antibody tests and increasing the number of vaccinated animals to eight. 

As shown in Table 2, none of the sera samples had detectable antibody titers measured by MAT and CFT before vaccination. Serum sample number 4860421 was CFT and MAT negative 15 days post-vaccination but transformed positive at day 30, also becoming positive for i-ELISA. Meanwhile, serum numbers 4860417, 4860422 and 4860423 transformed positive 30 days post-vaccination by i-ELISA. Serum sample number 4860419 showed a minimal MAT positivity at 30 days post-vaccination but remained negative by CFT and i-ELISA along the whole experiment.

**Figure 2:**
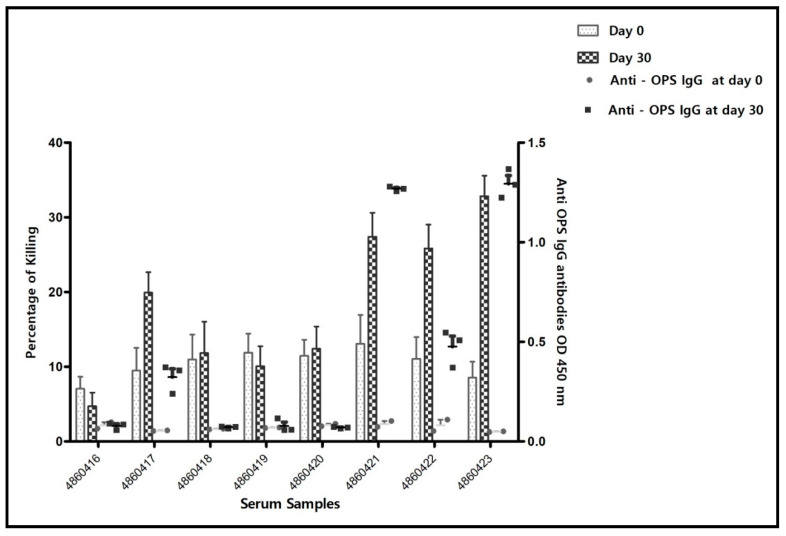
Percentage of cell killing of *B. melitensis* 16M cells by the individual serum samples on day 0, prior to vaccination and day 30 post-vaccination period, along with development of anti-*Brucella OPS* i-ELISA titers respectively (results obtained in the second set of experiments). i-ELISA results are of one experiment in triplicates. SBA results represent data of percentage of cell killing from 4 experiments, each including duplicates. Bars stand for standard error of mean.

**Figure 3:**
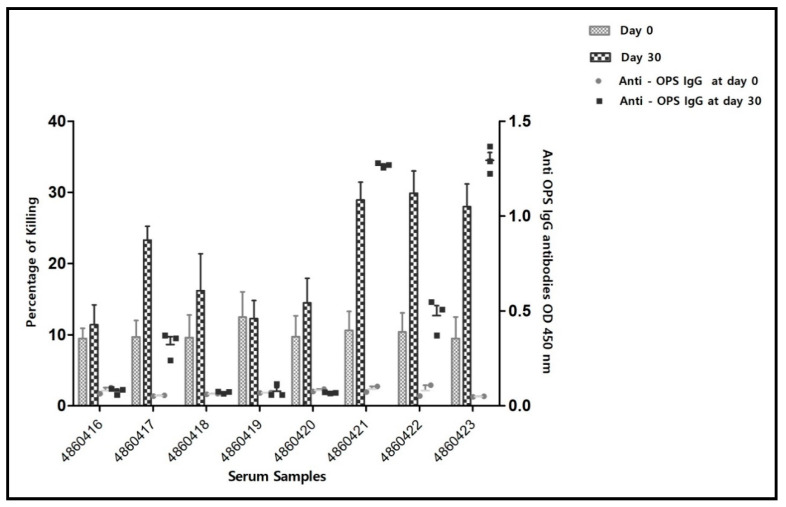
Percentage of cell killing of *B. melitensis* Rev.1 cells by the individual serum samples on day 0, prior to vaccination and day 30 post-vaccination periods, along with the development of anti-*Brucella*-OPS i-ELISA titers (results obtained in the second set of experiments). i-ELISA results are of one experiment in triplicates. SBA results represent data of percentage of killing from 4 experiments, each including duplicates. Bars stand for standard error of mean.

As shown in Table 2, CFT and MAT negative sera were still shown positive by i-ELISA, indicating the higher sensitivity of the i-ELISA method in comparison to the standard serology. In fact, CFT and SAT have been adjusted a priori to be able to perform as qualitative DIVA methods which excludes reading of vaccine elicited antibodies. Figure 2 and Figure 3 show that serum samples before vaccination lacked anti-*Brucella* OPS antibodies but were endowed with a basic SBA level which varied among the serum samples between 8 to 13% of cell killing between strain 16M (Figure 2) and Rev. 1 (Figure 3). SBA against both Rev. 1 and 16M strains rose up among serum samples number 4860417, 4860421, 4860422 and 4860423 which significantly correlated with development of IgG OPS anti-*Brucella* antibody titers in the same sera on day 30 post-vaccination (against 16M, *p* < 0.05, 4860417; *p* < 0.01 4860421, 4860422; *p* < 0.001, 4860423 and against Rev. 1 (*p* < 0.05, 4860417; *p* < 0.01, 4860421; *p* < 0.001, 4860422, 4860423), Bonferroni’s post hoc test. Supplementary Material Figure S3 summarizes the results of individual sera samples when combined into two groups, those which lacked i-ELISA titers and in correlation were not endowed with SBA in contrast to the other serum samples which possessed i-ELISA titers and were endowed with SBA, respectively. Bonferroni two-way ANOVA analysis showed the significant killing difference (*p* > 0.001) against *B. melitensis* 16M and *B. melitensis* Rev.1 with anti-*Brucella* OPS antibodies sera in comparison to sera-lacking anti-*Brucella*-OPS antibodies.

#### 3.1.3. Serology and Serum Bactericidal Activity (SBA) of Post-Infection Sera

**Figure 4:**
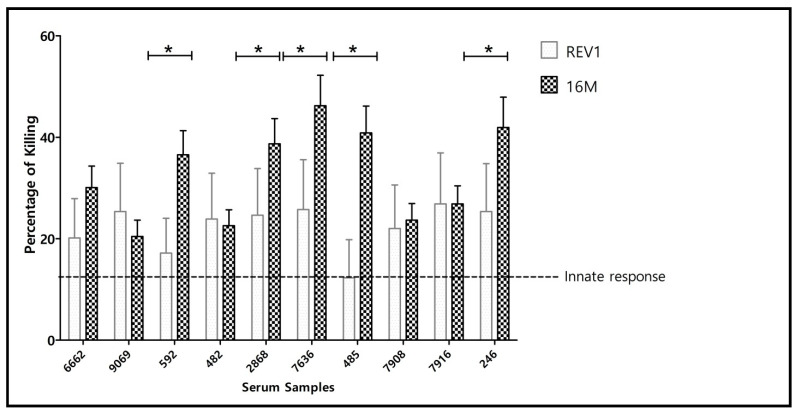
Percentage of cell killing of *B. melitensis* strain 16M and *B. melitensis* strain Rev. 1 Elberg by serum samples of infected sheep. Unpaired *T*-test shows overall significant difference between 16M and Rev. 1 cell killing (*p* < 0.05) (with Bonferroni correction for multiple comparisons), (* *p* < 0.05) shows the significant difference in the killing activity of individual sera. Horizontal line depicts percentage of *Brucella* cell killing attributed to innate immunity (A generalized innate killing activity of sheep serum samples calculated as a mean value of killing results of the four serum samples prior to animal vaccination). Killing activity above this level is considered, therefore, to be contributed by any of anti-*Brucella* antibodies present in the reaction, including non-anti-OPS antibodies.

We also examined SBA in serum samples obtained from *B. melitensis* naturally infected sheep, selecting animals according to their positive CFT serology of ≥1:20 which indicated presence of IgG anti-*Brucella* antibodies in the serum. Because animals in the field are identified bona fide, without information on their serum activity before exposure to *Brucella*, we used baseline SBA values from the SBA data mentioned above among sheep before vaccination Figure 1. As can be seen in Figure 4, serum samples of infected animals demonstrated complement mediated cell killing against both strains, all showing a higher cell killing activity than their baseline response (horizontal broken line). In this test, killing was more effective against strain 16M than against vaccine strain Rev.1 (unpaired *t*-test, *p* ≤ 0.05) reiterating the observation discussed in Figure 1.

## 4. Discussion

Use of Rev. 1 vaccine has always been conceived effective in protection against brucellosis due to elicitation of the cellular arm of the immune response. In contrast, development of antibodies has been considered as interfering with DIVA diagnosis of vaccinated herds [18,21,27,28]. This led scientists to favor the ocular application of the vaccine throughout vaccination campaigns, and our manuscript addresses this point by showing that ocular vaccination still elicits antibodies which are not detectable by standard serological tests, whereas LPS ELISA can identify them. We have hypothesized that anti-*Brucella*-OPS antibodies elicited following Rev. 1 sheep vaccination, or otherwise sheep infection, would be functional in host protection. To test this hypothesis, two sets of experiments were designed on the basis of studying development of anti-OPS antibodies by both CFT and MAT serology in the first set and anti-*Brucella*-OPS indirect ELISA serology in the other set. We have furthered the experiment by testing first development of SBA in correlation with Rev. 1 ocular vaccination and secondly, in sera obtained after natural *Brucella* infection. The serum agglutination test (MAT), complement fixation test CFT and i-ELISA were used to indicate presence of IgM (MAT) and IgG antibodies (CFT and i-ELISA) in the serum samples) [24].

Because Rev. 1 is delivered in Israel by the ocular route, aiming at reducing antibody titers, we specifically selected sheep that best represent their antibody response to Rev. 1 vaccination through CFT, MAT analyses in the first set of experiment and including i-ELISA in the second test, assuming i-ELISA would reveal anti-*Brucella*-OPS antibodies which MAT and CFT are missing, respectively. We chose two *B. melitensis* strains as read out analytes, a typical strain of the genus (*B. melitensis* strain 16M), and its counter live attenuated vaccine strain Rev.1 Elberg, passage 101, 1970, best representing the authentic Rev. 1 clone first developed by Elberg in the 1950s [29], aiming also at revealing how its attenuation has affected cell susceptibility to SBA.

From the Rev. 1 pre- and post-vaccination sera testing, our data have reiterated a past notion that anti-*Brucella* serum antibodies participate in *Brucella* cell killing. In this study we have clearly shown the correlation between development of elevated antibody levels, mostly IgG, against *Brucella*-OPS in sheep and percentage of cell killing in both cases of Rev. 1 ocular vaccination or following natural infection, respectively. We have observed, however, that in the vaccine experiment, CFT-positive serum samples showed significantly lower SBA against Rev. 1 cells than against strain 16M (Figure 1, *p* < 0.001; Bonferroni post hoc test) possibly explained by differences in the cell envelop between the vaccine strain and 16M, a typical strain of the genus [30]. One may thus take it one step further to hypothesize that vaccine strain Rev. 1 attenuation may have involved changes in its OPS structure.

Comparing between vaccine- and infection-induced antibodies provided a unique opportunity to test the functional responsiveness of the immune response against both *Brucella* strains. *B. melitensis* is naturally affiliated to small ruminants and *B. abortus* to cattle. Nevertheless, both strains share cross reactive antibodies against their OPS antigen making serology irrelevant in determining which was the etiological agent involved in an infection. Despite using the ocular vaccine, aimed at reducing the humoral response of the immune system, antibodies are developed by the host. 

We demonstrate that anti-*Brucella*-OPS antibodies induced by vaccination or natural infection are functional and may contribute to preventing infection by activating a complement mediated cell-killing mechanism. Until now, development of anti-*Brucella*-OPS antibodies has been considered a flaw in the vaccination policy due to hampering DIVA serological surveillance studies following Rev. 1 vaccination campaigns. To overcome this problem, enforcing a stringent vaccination policy where only young replacement animals are vaccinated by a single-lifetime inoculation and flock coverage is confirmed would be necessary.

Similar to Rev. 1 vaccination, our results now show that natural infection also accounts for development of functional anti-*Brucella* OPS antibodies that participate in SBA against *Brucella* (Figure 4). However, in the case of infection it is understood that the field strain persists in the flock and animal culling is required to stop the spread of the disease to other animals.

Our results indicate an opportunity to improve vaccination effectiveness against brucellosis by increasing development of anti-*Brucella*-OPS bactericidal antibodies using a DIVA established vaccine, first proposed by Bundle and McGiven (18). It has been shown that conjugate vaccines against other bacterial pathogens such as *Hemophilus influenzae* type b, *S. pneumoniae* or *Shigella* can induce stronger binding and functional antibody responses as compared to the corresponding natural infections [31,32,33]. The development and use of a conjugate *Brucella* vaccine should, therefore, be sought as an ultimate solution to maintaining high coverage of herd immunity. Rev. 1 vaccination of replacement animals should nevertheless remain a major strategic tool in controlling the disease by strengthening individual immunity among the animals through prevention of abortions thus minimizing the risks of building up chronic brucellosis in the flock.

## 5. Conclusions

Rev. 1 vaccination as well as *B. melitensis* infection elicit functional anti-*Brucella*-OPS antibodies which kill *Brucella* cells in vitro by the classical complement mediated pathway. Rev. 1 vaccine, which is a live attenuated strain, survives in the host for a critical period required for the buildup of the adapted cellular arm of the immune response. However, this vaccine does have adverse effects; it causes abortions if used in pregnant ewes, it elicits persistent antibody titers which hamper DIVA diagnosis and it is zoonotic.

In view of our results, we assume that endowing the host with anti-*Brucella*-OPS antibodies could augment the innate immune response against brucellosis, therefore aiding in protection against invading *Brucella* organisms at earlier phase of infection. Whereas natural development of anti-*Brucella*-OPS antibodies is associated with a T-cell-independent pathway, using a conjugate vaccine which relies on construction of a smooth OPS antigen bound to a protein carrier would provide a solution to constructing a safe, acellular vaccine important to development of herd immunity.

## Figures and Tables

**Table 1:** Anti-*Brucella*-OPS serum titers depicted by MAT and CFT in the first vaccine experiment before and after ocular vaccination with *B. melitensis* Rev. 1 vaccine.

Serum Identity Number	Before Vaccination (Day 0)		15 Days Post Vaccination (Day 15)		30 Days Post Vaccination (Day 30)	
	MAT	CFT	MAT	CFT	MAT	CFT
9912	Neg	Neg	Neg	Neg	1:80 (+3)	Neg
9913	Neg	Neg	1:40 (+3)	Neg	1:80 (+3)	1:5 (+3)
9914	Neg	Neg	Neg	Neg	Neg	Neg
9921	Neg	Neg	1:40 (+3)	Neg	1:80 (+3)	1:20 (+3)

Neg stands for Negative.

**Table 2:** Anti-*Brucella*-OPS serum titers depicted by three serological tests in the second vaccine experiment before and after ocular vaccination with *B. melitensis* Rev. 1 vaccine.

Sheep Number	Serology Results								
	Day 0 (19 July 2021)			Day 15 (3 August 2021)			Day 30 (19 August 2021)		
	SAT	CFT	ELISA IgG (OD)	SAT	CFT	ELISA (OD)	SAT	CFT	ELISA IgG (OD)
4860416	Neg	Neg	0.0805	Neg	Neg	0.077033333	Neg	Neg	0.078
4860417	Neg	Neg	0.053	1:20 (+4)	Neg	0.056	1:20 (+4)	Neg	0.323
4860418	Neg	Neg	0.062	Neg	Neg	0.054	1:20 (+4)	Neg	0.071
4860419	Neg	Neg	0.069	Neg	Neg	0.058	1:40 (+1)	Neg	0.077
4860420	Neg	Neg	0.082	Neg	Neg	0.056	Neg	Neg	0.0698
4860421	Neg	Neg	0.088	Neg	Neg	0.053	1:80 (+1)	1:10+	1.2682
4860422	Neg	Neg	0.081	Neg	Neg	0.049	1:20 (+4)	Neg	0.4757
4860423	Neg	Neg	0.049	Neg	Neg	0.054	Neg	Neg	1.2935

Neg stands for negative.

## Supplementary Materials

The following supporting information can be downloaded at: https://www.mdpi.com/article/10.3390/vaccines10020317/s1, Figure S1: Percentage of *B. melitensis* strain 16M cell killing by the individual serum samples; Figure S2: Percentages of *B. melitensis* Rev. 1 Elberg cell killing by the individual serum; Figure S3: Arithmetic mean value of *Brucella* cell killing efficiency by a combined pool of the 8 serum samples prior to vaccination and at day 30 in correlation with the presence of anti-*Brucella* OPS i-ELISA antibodies.
