# Supplementary material for: Natural Brucella melitensis Infection and Rev. 1 Vaccination Induce Specific Brucella O-Polysaccharide Antibodies Involved in Complement Mediated Brucella Cell Killing
Source: Vaccines (Basel). 2022 Feb 17;10(2):317. doi: 10.3390/vaccines10020317 (PMC8878583; doi:10.3390/vaccines10020317)
Supplement: Supplementary file 1 [file vaccines-10-00317-s001.zip › vaccines-1545915-supplementary.pdf]

vaccines

MDPI

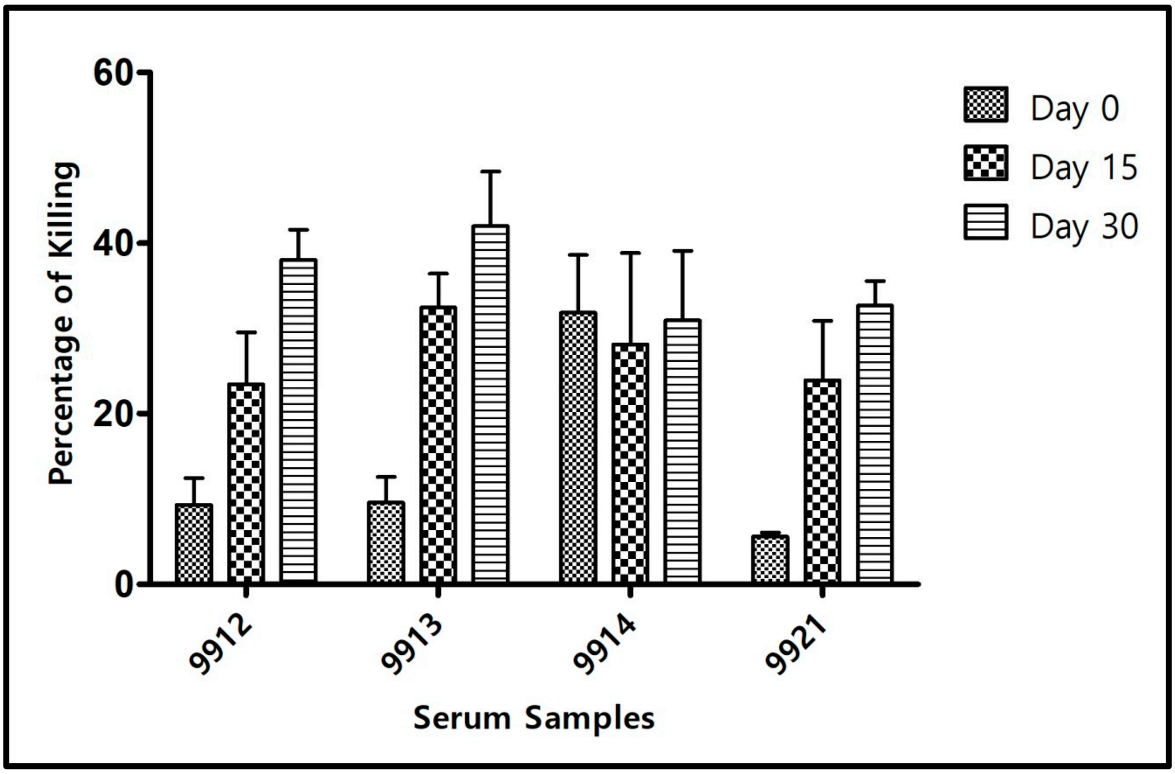

Figure S1. Percentage of *B. melitensis* strain 16M cell killing by the individual serum samples; day 0, prior to vaccination, day 15 and day 30 post-vaccination periods, respectively. (The results represent data of percentage of killing from 2 experiments, each including quadruplicates. ± stands for standard deviation.

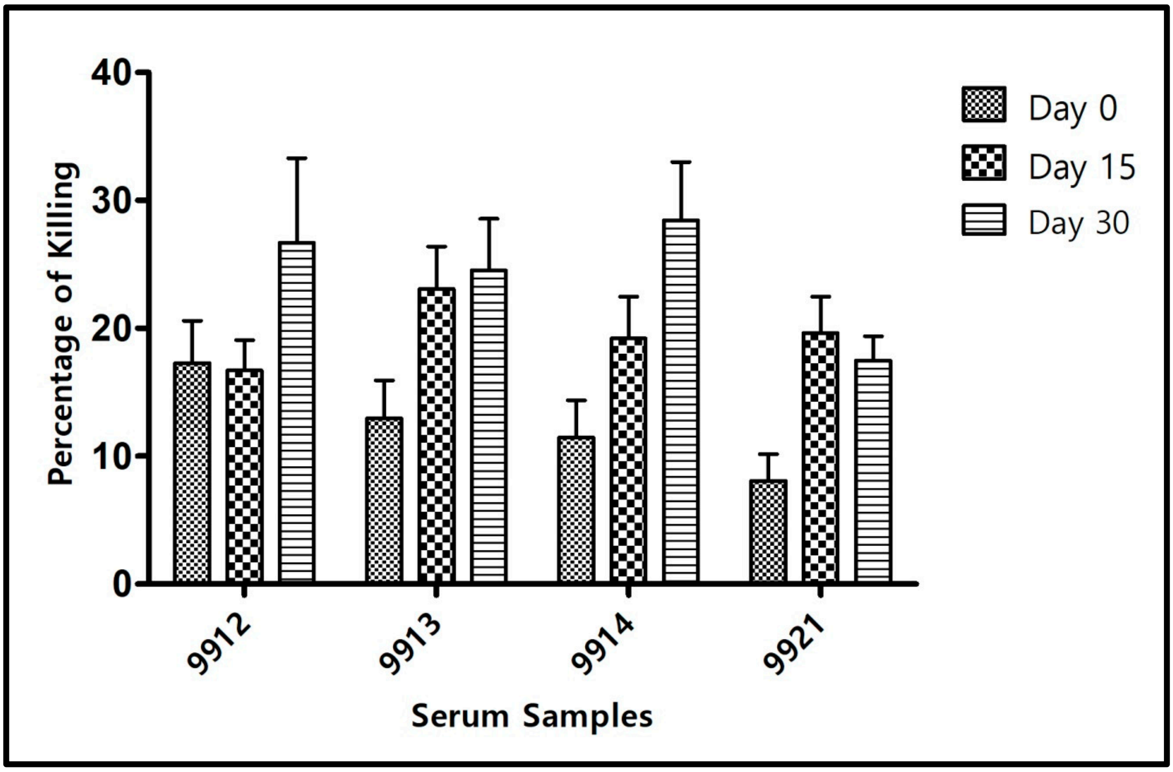

Figure S2. Percentages of *B. melitensis* Rev. 1 Elberg cell killing by the individual serum samples; day 0, prior to vaccination, day 15 and day 30 post-vaccination periods, respectively. The results represent data of percentage of killing from 2 experiments, each including quadruplicates. ± stands for standard deviation.

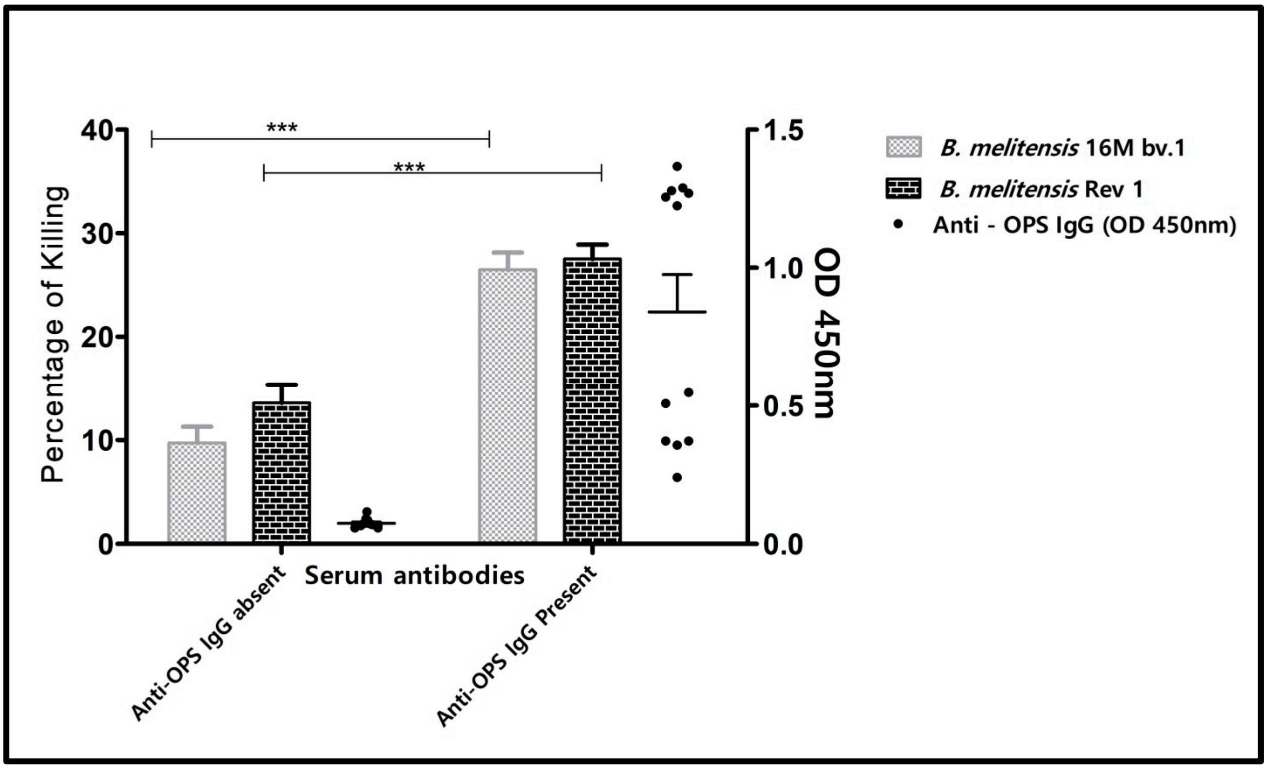

Figure S3. Arithmetic mean value of *Brucella* cell killing efficiency by a combined pool of the 8 serum samples prior to vaccination and at day 30 in correlation with the presence of anti – *Brucella* OPS i-ELISA antibodies. Two-Way ANOVA unweighted analysis of the mean values depicted the significance of cell killing (***,p>0.001) against both *Brucella* species by the sera having anti – *Brucella* OPS antibodies in comparison to sera before vaccination lacking anti – *Brucella* OPS antibodies. Through unpaired two tailed t-test there is significant difference (p<0.001) of anti – *Brucella* OPS IgG antibodies between the two groups.
